# The Relationship Between Serum Lipids and the Formation of Colorectal Polyps

**DOI:** 10.7759/cureus.57511

**Published:** 2024-04-03

**Authors:** Yiğit Düzköylü, Mahmut Kaan Demircioğlu, Hüseyin Kılavuz, Serkan Sari

**Affiliations:** 1 Gastroenterological Surgery, Başakşehir Çam and Sakura City Hospital, İstanbul, TUR; 2 Surgical Oncology, Umraniye Training and Research Hospital, İstanbul, TUR; 3 General Surgery, Başakşehir Çam and Sakura City Hospital, İstanbul, TUR

**Keywords:** colorectal cancer, low-density lipoprotein cholesterol, triglyceride, total cholesterol, hyperlipidemia, colorectal polyp

## Abstract

Background and aims: Obesity, metabolic syndrome, and hyperlipidemia are known as risk factors for colorectal tumors. Colorectal polyps are accepted as potential precursors of colorectal cancer (CRC). This study was designed to clarify the association between the levels of serum lipids and the presence of colorectal polyps.

Methods: This study was conducted at Basaksehir Cam and Sakura City Hospital, Gastroenterological Surgery Clinic, Istanbul, Turkey. We retrospectively analyzed patients who underwent colonoscopy with serum lipid profile within one month for a one-year period. Groups were analyzed in terms of the correlation between hyperlipidemia and the formation of polyps. The study group was also evaluated in terms of the polyp type, localization, and number.

Results: Among 453 patients, females were 248 and males were 211, with a mean age of 56.7. The study and control groups involved 259 and 194 patients, respectively. The age and serum levels of low-density lipoprotein cholesterol (LDL-C), total cholesterol (TC), and triglyceride (TG) were found to be statistically significant in terms of polyp presence and number (p < 0.05).

Conclusion: Colorectal polyps are well-known precursors of CRC. We found that the combination of elevated serum levels of low-density lipoprotein cholesterol, total cholesterol, and triglycerides may be a risk predictor for the presence of colorectal polyps, which can be advantageous in cancer screening.

## Introduction

Colorectal cancer (CRC) is the fourth most common cause of cancer-related death and the third most common cancer worldwide [1,2]. Colorectal polyps are thought to be potential precursors of CRC that develop on the mucosal layers of the colon [3] and grow into colorectal adenomas. In their recent study, Zhao et al. showed that 80% of adenomas are responsible for CRC progression [4]. Risk factors associated with polyp formation include villous component or high-grade adenomas and large or multiple polyps [5]. According to the World Health Organization (WHO) classification, polyps are divided into adenomatous, inflammatory, hyperplastic, and hamartomatous types [6]. Adenomatous polyps have different types, such as tubular, villous, and tubulovillous adenomas [7]. All types of polyps have an incidence rate of 1-43% [8] and have the potential to grow into CRC, which is known as the adenoma-carcinoma pathway in the development of CRC [9].

Especially after recent technological developments, colonoscopy with high-definition equipment in the hands of experienced professionals has the potential to detect, diagnose, and treat adenomas and even early-stage colorectal tumors [10]. Thanks to this ability of colonoscopic procedures, the endoscopic resection of colorectal polyps has led to a significant reduction in the mortality rate of CRC [5]. Unfortunately, tumor recurrence is a major complication following local and minimally invasive resections [11]. Despite the advantageous ability of colonoscopy to detect premalignant polyps, a gold standard surveillance in terms of both mortality and social resources that is generally suitable for public health systems has not yet been described. Therefore, identifying risk factors for polyp formation in terms of premalignant signs may help to improve the design of screening surveillance and patient outcomes [12].

Metabolic syndrome has been implicated as a risk factor for polyp formation through its relationship with gut microbiota and inflammatory pathways [13]. It is now known that hyperinsulinemia with obesity, which are also elements of the metabolic syndrome, are risk factors for CRC along with hyperglycemia and higher body mass index (BMI) [10,13,14]. However, despite being an important component of metabolic syndrome and obesity, the role of serum lipids in the development of colorectal polyps has not been well described. Although published results are controversial, low levels of high-density lipoprotein cholesterol (HDL-C) and high levels of triglycerides, total cholesterol (TC), and low-density lipoprotein cholesterol (LDL-C) are accepted as risk factors for adenomas [15,16]. Conversely, several studies with large groups have failed to show an association between LDL-C and colorectal lesions [17,18], but we believe that further studies should be performed for evidence-based analyses because of the facts showing the relationship between lipid metabolism in tissues and chronic inflammatory pathways [19]. Among these, a recent study by Macarie et al. showed that hyperlipidemia is an independent risk factor for premalignant polyps [20]. The studies supporting a positive association between higher serum lipid levels and polyp formation and recurrence suggest two possible underlying mechanisms: The first mechanism is that insulin resistance increases the transformation of colorectal polyps into premalignant types and recurrence rates by inhibiting apoptosis and enabling the expression of insulin-like growth factor-1 (IGF-1) [21]. The second possible mechanism is the disruption of the bile acid cycle by lipid abnormalities [22]. In support of these data, Siddiqui et al. in their study of the effects of cholesterol-lowering drugs showed that statins reduced the formation of adenomatous polyps and CRC in long-term use [23].

In our study, we aimed to evaluate the relationship between levels of serum cholesterol, LDL-C, HDL-C, and TG and increase in the formation of colorectal polyps, compared with control patients. Based on the study group, we also aimed to determine a possible relationship among polyp types and localization to contribute to the existing literature.

## Materials and methods

After obtaining local ethics committee approval, we designed our retrospective study in Basaksehir Cam and Sakura City Hospital, Gastroenterological Surgery Clinic, a single tertiary center in Istanbul, Turkey. We studied the patients who underwent colonoscopy within one year (January 2022-January 2023). Patients were included if their serum lipid profile was approved within one month prior to colonoscopy. Exclusion criteria were previous gastrointestinal surgery, including microinvasive bariatric procedures, severe systemic comorbidities, diagnosis of gastrointestinal tumors, incomplete or suboptimal endoscopic evaluation, and chronic use of lipid-lowering drugs. As this was a retrospective study, diet, lifestyle, and genetic factors could not be assessed. A total of 453 patients were included in the study. The study group consisted of patients with histopathologically confirmed colorectal polyp excisions, while the control group consisted of patients without any findings of colorectal polyps. The groups were compared in terms of demographics, serum lipid profile, and BMI and their effect on the presence of colorectal polyps. Patient demographics, BMI, and lipid profile were analyzed for their statistical effect on polyp type, location, and number. Colonoscopy procedures were performed after a liquid-based diet one day prior to the examination. Polyethylene glycol and rectal enema were used for bowel preparation. A clear examination of all segments of the colon, including the caecum, was considered an optimal procedure, and the patient was enrolled in the study. All procedures were performed by experienced endoscopists in a single tertiary center. Histopathological features of polyps were analyzed retrospectively from pathology reports. Polyp types were classified as hyperplastic (HP), tubular adenoma (TA), tubulovillous adenoma (TVA), high-grade dysplasia (HGD), and malignant polyps. Polyps had been excised with simple excision using standard endoscopic forceps or with endoscopic mucosal (EMR) and submucosal dissection (ESD) depending on the size and morphology of the polyp. Lipid profile tests included TC, LDL-C, HDL-C, and TG, with the addition of fasting serum glucose (FSG), which can affect the serum levels of lipids in the presence of diabetic dyslipidemia. All serum lipid parameters were analyzed in the biochemistry laboratory of the hospital with the same type of kit.

Statistical analysis

Data are shown as mean ± standard deviation and median (minimum-maximum) variables. Comparison with normal dispersion was performed with Kolmogorov-Smirnov and Shapiro-Wilk tests. In the statistical analyses, the T test, Mann-Whitney U test, and Kruskal-Wallis test were performed for independent variables. Bonferroni-corrected Mann-Whitney U test was used for post hoc tests. A p-value < 0.05 was considered statistically significant. IBM SPSS Statistics for Windows, version 25.0 (released 2017, IBM Corp., Armonk, NY) was used for the statistical study.

## Results

A total of 453 patients were included in the study; females were 248 and males were 211, with a mean age of 56.7. The study and control groups involved 259 and 194 patients, respectively. The age, BMI, FSG, and serum lipid profiles of the 453 patients are shown in Table 1.

**Table 1:** Age, BMI, and lipid profile of the patients SD: standard deviation, FSG: fasting serum glucose, BMI: body mass index, TC: total cholesterol, TG: triglyceride, HDL-C: high-density lipoprotein cholesterol, LDL-C: low-density lipoprotein cholesterol

Variables	Mean ± SD
Age	56.7 ± 13.5
FSG (mmol/L)	106.4 ± 40.1
BMI	28.1 ± 4.8
TC (mg/dL)	183.2 ± 45.9
TG (mg/dL)	149.6 ± 88.4
HDL-C (mg/dL)	47.01 ± 14.6
LDL-C (mg/dL)	105.7 ± 37.6

Based on the statistical analysis, age and serum levels of LDL-C, TC, and TG were found to be statistically significant in terms of polyp presence. The variables are shown in Table 2.

**Table 2:** Variables affecting polyp presence *: Mann-Whitney U test, +: Presence of polyp, -: abscence of polyp, FSG: fasting serum glucose, BMI: body mass index, TC: total cholesterol, TG: triglyceride, HDL-C: high-density lipoprotein cholesterol, LDL-C: low-density lipoprotein cholesterol

Variables	Polyp presence		p^*^
	+	-	
	Median (Min-Max)		
Age	55 (18-89)	61 (30-92)	<0.001
FSG (mmol/L)	93 (54-397)	96 (54-370)	0.063
BMI	28.15 (17.8-50.7)	28 (18.2-43)	0.713
TC (mg/dL)	174 (67-371)	187 (72-330)	0.001
TG (mg/dL)	120.5 (43-604)	129 (38-673)	0.031
HDL-C (mg/dL)	47 (11-97)	44 (16-105)	0.059
LDL-C (mg/dL)	93 (16-258)	112 (22-225)	<0.001

Among 259 patients in the study group, polyps were classified into hyperplastic (HP), tubular adenoma (TA), tubulovillous adenoma (TVA), high-grade dysplasia (HGD), and malignant polyps. TA was the most common type in the study group (n = 151, 58.3%), followed by hyperplastic polyps. Malignancy was encountered in 11 patients (4.24%). The same variables were analyzed in terms of polyp types, and no statistically significant result was obtained following the Mann-Whitney U test, except that age was a significant variable in hyperplastic polyps according to the T-test.

In the study group, the location of polyps was analyzed, and the sigmoid colon was found to be the most commonly affected segment, followed by the rectum and descending colon. The number of polyps was analyzed with the same variables. Age, LDL-C, and TC were found to be statistically significant in terms of the higher polyp number. The results are shown in Table 3.

**Table 3:** Variables affecting the polyp number *: Variables are shown as median (min-max). **: a: different when compared to one group, b: compared to two groups, c: compared to more than two groups. ***: Kruskal-Wallis test FSG: fasting serum glucose, BMI: body mass index, TC: total cholesterol, TG: triglyceride, HDL-C: high-density lipoprotein cholesterol, LDL-C: low-density lipoprotein cholesterol

Variable*	Polyp number**				p^***^
	None	1	2	>2	
Age	55 (18-89)	58 (30-92)	59 (31-80)	63.5 (35-78)	<0.001
FSG (mmol/L)	93 (54-397)	97 (54-370)	93 (63-252)	95 (67-311)	0.08575
BMI	28.2 (17.8-50.7)	27.7 (18.8-43)	28.7 (20.1-43)	26.65 (18.2-41.6)	0.26904
TC (mg/dL)	174 (67-371)	187 (72-307)	182 (116-276)	193.5 (106-330)	0.00231
TG (mg/dL)	121 (43-604)	134 (38-673)	114 (42-363)	117.5 (52-423)	0.14994
HDL-C (mg/dL)	47 (11-97)	45 (16-103)	43 (16-105)	45 (26-94)	0.17292
LDL-C (mg/dL)	93 (16-258)	111 (22-209)	107 (46-170)	122 (44-225)	<0.001

## Discussion

The relationship between the formation of colorectal polyps and serum lipid profile has attracted attention in recent years and has been evaluated in randomized controlled trials with large study and control groups. The rationale for investigating various premalignant signs and markers of CRC may be due to the increasing mortality from CRC, which can be prevented by early diagnosis. In addition, colorectal polyps, which are known precursors of CRC, can be easily detected and treated. Endoscopic treatment of premalignant polyps is crucial for reducing CRC-related mortality, but the selection of the most appropriate screening method and high-risk patients is still controversial based on published studies. Endoscopic resection of polyps has been shown to reduce CRC mortality [24]. Unfortunately, the results are still controversial. Some of the published studies have shown that serum TC and TG levels are significantly associated with the formation of colorectal polyps [10,25], while other studies have failed to show a similar association or even suggested an inverse association [26]. Obesity and high BMI are associated with an increased risk of polyp presence and recurrence after resection, thought to be due to increased levels of insulin resistance and IGF-1 [12,27]. Insulin resistance also increases TG and FSG levels with gluconeogenesis [28]. Dyslipidemia affects the microenvironment, leading to DNA damage, cell proliferation, and neoangiogenesis [29]. There are also studies showing a positive correlation between LDL-C and oxidative stress [30]. Several studies have shown that higher TG and lower HDL-C levels significantly increase the risk of colorectal adenomas [15,17]. Some researchers have even shown a direct relationship between elevated TG levels and CRC [20,31]. The underlying etiology is thought to be the activation of IGF-1 leading to inhibition of apoptosis and dyslipidemia leading to proinflammatory cytokines [18,32] and the accumulation of TG in intestinal cells [19].

These facts have led clinicians to evaluate serum levels of lipids and fasting glucose, which are directly related to obesity and metabolic syndrome, to determine a possible association with colorectal polyp formation. Stocks et al. in their Metabolic Syndrome and Cancer Project found that TG was associated with an increased risk of CRC [33]. In their study of 4,122 patients in the Chinese population, Liu et al. suggested that large waist circumference, low levels of HDL-C, and high levels of TG were significantly associated with colorectal adenoma formation [34]. In their systematic review and meta-analysis evaluating the results of studies that included East Asian populations for 14 years, Passarelli et al. showed that high levels of LDL-C and low levels of HDL-C were associated with the prevalence of colorectal adenomas [35]. In our study, we aimed to investigate the relationship between serum lipid profile and colorectal polyp formation, including polyp type, number, and location. We found a statistically significant relationship between colorectal polyps and serum lipids. Similar to most previous studies, our results showed that serum levels of LDL-C, TC, and TG were statistically significant for polyp presence (p < 0.05). We also found that the number of polyps was significantly correlated with the levels of LDL-C and TC (p < 0.05). In our study, the specificity of LDL-C was higher than that of TG, in terms of the p-value, which is contrary to the study by Xie et al. They also found that the combination of serum levels of TG and LDL-C were independent risk factors for the presence of colorectal polyps. In addition, they suggested that LDL-C levels were higher in the HP and TA groups [10]. Conversely, we could not find a significant result in the mean of polyp types. International guidelines have also shown that the prevalence of colorectal adenoma increases with age [36], which is consistent with our results showing a correlation not only with the presence of polyps but also with the number of polyps. There are studies suggesting a male predominance in the development of colorectal adenoma [37], but we did not find significant results for gender.

Our study has several limitations. The retrospective design of the study led us to evaluate the lipid profiles within one month of colonoscopy instead of performing biochemical tests and colonoscopy simultaneously. Second, the colonoscopic procedures were not performed by a single endoscopist, although all were experienced professionals. Third, although not significantly lower than in previous studies, our limited number of patients in the groups may have altered the statistical results, depending on the fact that some of our results were slightly above or below the statistically significant value.

## Conclusions

It is a well-known fact that hypercholesterolemia is among the underlying reasons for colorectal polyp development. Following the results of previously published literature, we found a statistically significant association between the presence of colorectal polyps, their number, and serum levels of LDL-C, TC, and TG in our study. Despite its retrospective design, our study may contribute to future studies in this field with expanded patient groups and meta-analysis studies, which may lead clinicians to be more cautious, especially in young people with dyslipidemia. Accordingly, it can contribute to colonoscopic screening for CRC and decreasing mortality rates.
